# Preclinical evaluation of a chemically detoxified pneumolysin as pneumococcal vaccine antigen

**DOI:** 10.1080/21645515.2016.1234553

**Published:** 2016-10-21

**Authors:** Philippe Hermand, Annick Vandercammen, Emmanuel Mertens, Emmanuel Di Paolo, Vincent Verlant, Philippe Denoël, Fabrice Godfroid

**Affiliations:** GSK Vaccines, Rixensart, Belgium

**Keywords:** Detoxification, mouse, pneumococcal antigen, pneumolysin, *Streptococcus pneumoniae*, vaccine

## Abstract

The use of protein antigens able to protect against the majority of *Streptococcus pneumoniae* serotypes is envisaged as stand-alone and/or complement to the current capsular polysaccharide-based pneumococcal vaccines. Pneumolysin (Ply) is a key virulence factor that is highly conserved in amino acid sesec-typsecquence across pneumococcal serotypes, and therefore may be considered as a vaccine target. However, native Ply cannot be used in vaccines due to its intrinsic cytolytic activity. In the present work a completely, irreversibly detoxified pneumolysin (dPly) has been generated using an optimized formaldehyde treatment. Detoxi-fication was confirmed by dPly challenge in mice and histological analysis of the injection site in rats. Immunization with dPly elicited Ply-specific functional antibodies that were able to inhibit Ply activity in a hemolysis assay. In addition, immunization with dPly protected mice against lethal intranasal challenge with Ply, and intranasal immunization inhibited nasopharyngeal colonization after intranasal challenge with homologous or heterologous pneumococcal strain. Our findings supported dPly as a valid candidate antigen for further pneumococcal vaccine development.

## Introduction

*Streptococcus pneumoniae* is responsible for a large spectrum of infections, including otitis media, meningitis and pneumonia.^1,2^ Current pneumococcal vaccines are based on pneumococcal capsular polysaccharides (PS) of the dominant disease-causing serotypes.^3-8^ They have greatly helped to reduce the burden of pneumococcal diseases, but there remains disease burden caused by serotypes not included in existing vaccines and the emergence of non-vaccine serotype(s) may ultimately reduce their overall effect.^9-11^ In the hope of circumventing the limitations of polysaccharide capsule-based vaccines, efforts are being made to evaluate the potential of common pneumococcal proteins for next generation products.^12^

Pneumolysin (Ply) is a ubiquitous virulence factor of *S. pneumoniae* showing cytolytic activity.^13^ This protein is released from the bacteria and its capacity to form pores in cholesterol-rich membranes causes severe tissue damage, which facilitates further colonization. It also activates complement,^14^ contributes to the inflammatory response of the infected individuals^15^ and plays an active role in acute lung injury.^16^ Recently, a new role was attributed to Ply in the development of biofilms.^17^ Ply was also shown to be involved in the mechanism of immunomodulation that allows the establishment of long term carriage.^18^ Preclinical reports have shown the importance of Ply in pneumococcal infection by investigating native Ply-deficient mutants,^19,20^ and others highlighted the protective role afforded by Ply-specific antibodies.^21,22^ Such a protective role was not only observed in experimental animal models, but also seems to be supported by human data.^23^ In addition to being recognized as a virulence factor, Ply shows a highly conserved amino acid sequence across strains,^24^ which makes it an attractive vaccine antigen candidate. Ply was already considered for vaccination 30 y ago,^25,26^ but the native protein could not be used due to its intrinsic cytolytic activity.^27-29^ Designing a Ply candidate vaccine antigen with the appropriate detoxification and immune profile was challenging. First, site-directed mutagenesis was used to generate Ply mutants with reduced hemolytic activity.^20,30-34^ More recently, a rationally *in silico* designed non-toxic Ply mutant^35^ was shown to induce neutralizing antibodies that protect against pneumonia^22^ and to elicit functional Ply-specific antibodies in a phase 1 clinical trial.^36^

We have investigated an alternative method of producing detoxified pneumolysin (dPly), which consists of abolishing the toxic activity of Ply by formaldehyde treatment. This method also aimed to yield a more stable antigen than native Ply and is compatible with large-scale manufacturing. Here, we report on early preclinical studies, particularly the production method of dPly, its characterization, as well as its potential to be incorporated in pneumococcal vaccines as evaluated in animal challenge models.

## Results

### Characterization of dPly

**Figure 1.**
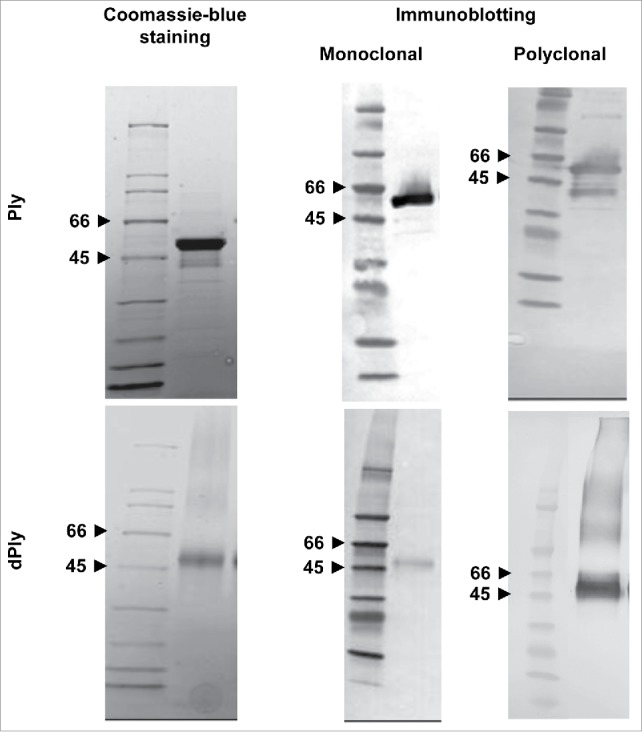
SDS-PAGE analysis of recombinant Ply (Ply) and dPly. Purified recombinant Ply and dPly were analyzed in Coomassie blue-stained SDS-PAGE gel, run in reducing conditions. Gel was also blotted and the membranes probed with in-house monoclonal or polyclonal anti-Ply antibodies.

The dPly molecule was examined in Coomassie blue-stained sodium-dodecyl-sulphate (SDS) polyacrylamide gel in reducing conditions, with Ply as a comparator (Fig. 1). Both Ply and dPly appeared as a major band at around 50-55 kDa, which corresponds to the molecular mass of native Ply. Blotting and probing with monoclonal anti-Ply antibody confirmed the nature of the protein bands. Although immunoblotting is not a quantitative method, one may notice that the staining of the band was fainter in the case of dPly, which may indicate that the specific epitope recognized by the monoclonal antibody was somehow masked by the formaldehyde treatment, but staining was sufficient to characterize the Ply nature of the band. In contrast, there was no difference in band intensity when probing with polyclonal antibodies. In addition, with anti-Ply polyclonal antibodies, high molecular weight species were visible, which we attributed to the presence of multimeric pneumolysin due to some formaldehyde inter-molecular cross-linking.

### Analysis of dPly demonstrates no residual toxicity

**Figure 2.**
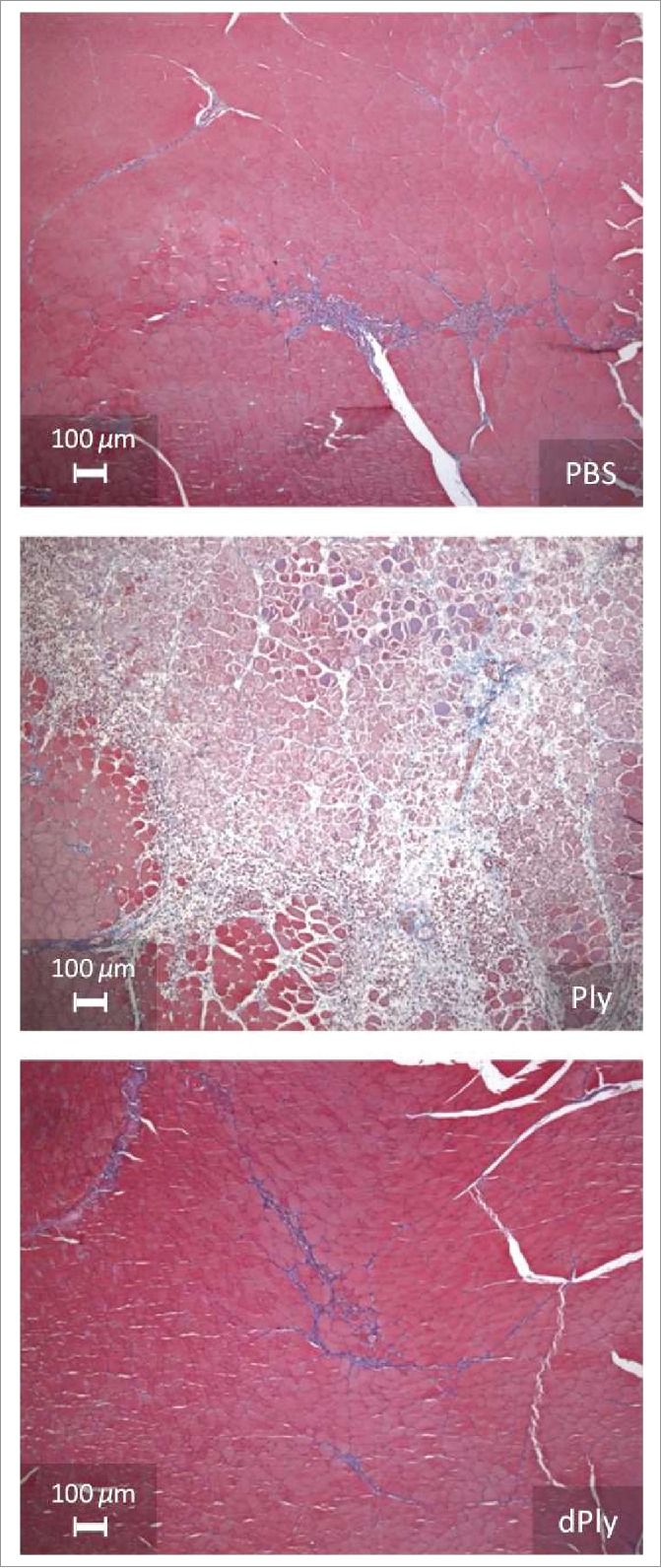
Microphotographs showing the injection site after intramuscular injection of either phosphate-buffered saline, Ply (10 µg) or dPly (10 µg) in rat tibialis.

Two different models were used to determine whether dPly had residual cytolytic activity. First, local reactogenicity was evaluated in rats by histo-pathological examination after intra-muscular injection (Fig. 2). Three days after injection of Ply, moderate to marked muscular fiber necrosis/degeneration associated with moderate inflammatory process and slight hemorrhage were observed. In contrast, injection of dPly induced minimal muscular alterations, similar to those observed with phosphate-buffered saline (PBS). These alterations were characterized by minimal degeneration/regeneration process associated with minimal inflammation and were related to needle track traumatism.

Potential residual toxicity was also evaluated in a mouse model of intranasal lethal challenge. In this model, 100% of mice (10/10) died within 3 d after intranasal deposition of 10 µg Ply, whereas all mice survived after 50 µg dPly, a dose corresponding to 70-fold the LD_50_ of native Ply. In addition, the hemolytic activity of dPly was shown to be 10^7^ times lower than that of Ply, below the assay limit of detection. Thus, dPly can be considered as completely inactive, outperforming in this regard the first generation of mutated Ply's,^37^ and being at least equally performant as the most recent Ply mutants.^35^

As a comparison, the detoxified Ply produced using the least effective detoxification condition during the pilot phase still was more than 10,000-fold less active than the recombinant Ply and was not lethal to the challenged animals.

### dPly is a stable antigen

Experiments were performed to evaluate whether dPly was stably detoxified. To this end, the antigen was stored at 37°C for up to 35 d before determination of its hemolytic activity. Irreversibility of detoxification was a criterion to select the optimal reaction conditions during the pilot phase. Detoxified Ply's obtained using non-optimal detoxification conditions were shown to recover some hemolytic activity during storage. Typically, reversal of hemolytic activity reached a plateau within the first 2 weeks of storage. In contrast, optimally detoxified Ply did not recover hemolytic activity after storage for up to 35 d at 37°C.

In another, *in vivo*, stability experiment, dPly was stored at 37°C for up to 44 d. In this experiment, 50 µg stored dPly was intranasally given to 10 mice and all of them survived the challenge, indicating that the antigen did not reacquire toxicity.

Altogether, these results indicate that the covalent links formed by formaldehyde treatment in the selected experimental conditions eliminated Ply cytolytic activity and were stable upon storage.

### Immunization with dPly generates functional anti-Ply antibodies

**Figure 3.**
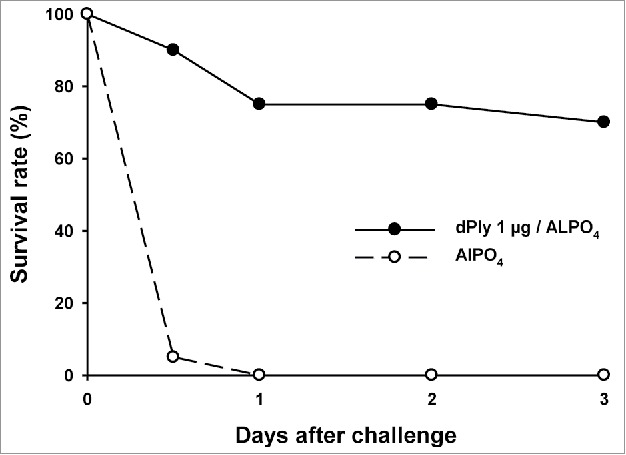
Mouse survival upon pneumolysin intranasal challenge. Mice (n = 20/group) were immunized twice intramuscularly at a 2-week interval with 1 µg of dPly adjuvanted with alum or with alum only. Fourteen days after the second injection, mice were challenged intranasally with 2 µg of Ply. The mortality was recorded during 3 d.

Immunization of mice with 1 µg dPly, adjuvanted with AlPO_4_ induced anti-Ply IgG antibodies, as measured by enzyme-linked immunosorbent assay (ELISA). Seroconversion was 100% and the mean anti-Ply IgG concentration was 110.8 µg/mL (95%CI: 77.93-157.6). The biological functionality of the generated anti-Ply antibodies was analyzed *in vitro* by hemolysis inhibition assay. For that, pooled serum dilutions were pre-incubated with Ply prior to hemolysis assay. The hemolysis mid-point titers were 50 for the control group immunized with adjuvant only and 300 for the group immunized with 1 µg dPly, showing that the antibodies generated by dPly immunization were able to inhibit the toxic activity of Ply. In addition, the functionality of the generated anti-dPly antibodies was evaluated *in vivo* in a mouse Ply lethal intranasal challenge model. In this model, vaccination with dPly allowed the survival of 70% of mice at Day 3 after homologous challenge with Ply, while all animals had died by Day 1 in the control group (p < 0.001; Fig. 3).

### Immunization with dPly protects against naso-pharyngeal colonization

**Figure 4.**
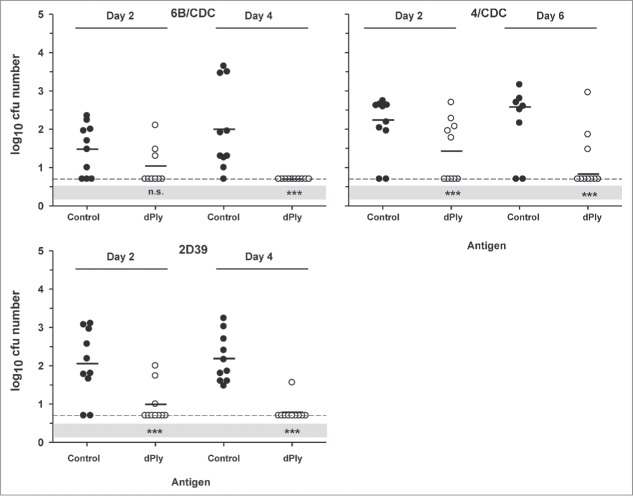
Vaccine efficacy in a *S. pneumoniae* nasopharyngeal colonization model. Mice were immunized intranasally with adjuvanted dPly or adjuvant alone (control) before they were intranasally challenged with either the pneumococcal strain 6B/CDC, 2D39 or 4/CDC. Bacterial colonies in nasal washings were counted at day 2 and at day 4 (day 6 for 4/CDC) post-challenge and expressed as log_10_ mean CFU. Each dot represents a mouse. Dashed lines indicate the limit of detection (at 0.70); Black horizontal bars are geometric means. Statistical analyses were carried out per day with ANOVA. All significant differences, compared with the control, are shown. ***, P< 0.001, n.s., not significant.

The effects of immunization with dPly were also analyzed in a mouse model of nasopharyngeal colonization. After immunization, intranasal challenges were made with 2 homologous (2/D39 and 6B/CDC carrying Ply allele 1) and one heterologous (4/CDC, carrying Ply allele 2) strains. The number of CFUs was measured on 2 time points. Our results showed that immunization with dPly afforded protection against the 3 strains (Fig. 4). Two days after challenge the number of CFUs was lower in the dPly-immunized group, compared with control (p < 0.001). This was not the case for the 6B/CDC challenge, but this seemed to be due rather to a lower colonization rate in the control mice than to a weaker effect of dPly immunization. After four or 6 days, the number of colonies was lower in the dPly groups (p < 0.001) independently of the strain. However, 3 dPly-immunized mice remained colonized by 4/CDC strain and one by 2D39.

## Discussion

Several pneumococcal proteins have been considered as possible antigen for next generation pneumococcal vaccines. However, few combine high immunogenicity and protection with good conservation across the various pneumococcal strains. Ply is a key virulence factor conserved across the *S. pneumoniae* strains, which makes it an attractive candidate for pneumococcal protein vaccine development. However, Ply is toxic, exhibiting cytolytic activity, and thus cannot be used for vaccination in its native form.

The main objective of this work was to obtain a fully detoxified molecule that does not revert to toxicity and retains antigenicity. Here, detoxification of Ply was achieved by formaldehyde treatment. Chemical detoxification was evaluated in this work, as this technique has been successfully applied to several bacterial toxins which are now used in well-established vaccines. Formaldehyde treatment requires a balance between antigenicity and residual toxicity. Heavy treatment yields a fully detoxified molecule but with low antigenicity, whereas too light a treatment may result in a molecule potentially able to revert to the active form. Formaldehyde reacts with proteins through a large panel of reactions involving various amino acids. Stable cross-linking with formaldehyde is a 2-phase reaction.^38^ In the first phase, formaldehyde reacts rapidly with primary amines, forming Schiff bases. These links are easily reversible; hence the second phase is needed to obtain irreversible links. During this second phase, the Schiff bases react with amino acids, particularly lysine, tryptophan, histidine and tyrosine, to form stable, irreversible links. An extended incubation time was required to warrant the irreversibility of detoxification. Moreover, the addition of free lysine in the incubation medium, as extra substrate to interact with Schiff bases, contributed to the complete conversion of the Schiff bases into stable links. Free lysine in the medium was also meant to limit formation of Ply intermolecular links, which may otherwise lead to Ply aggregation. In pilot experiments, several conditions of incubation time, pH, formaldehyde and lysine concentrations were evaluated to identify conditions leading to a stable, complete, irreversible and reproducible detoxification. The selected formaldehyde treatment conditions were 21 d at 40°C in potassium phosphate 25 mM, pH 7.0, containing formaldehyde 0.1% (w/v) and lysine 50 mM. In addition to being irreversibly detoxified in these conditions, dPly was also shown not to be degraded or aggregated upon long-term storage, whereas non chemically-modified Ply's, such as native Ply and recombinant Ply are known to readily aggregate in solution. The formaldehyde treatment thus seems to have stabilized this antigen, as has been observed with other bacterial toxins.^39^

After demonstrating the detoxification and stability of dPly, we aimed to evaluate with animal challenge models its potential as a vaccine candidate. Immunization of mice with dPly was shown to elicit functional anti-Ply antibodies able to inhibit Ply-induced hemolysis. It must be emphasized that Ply being a toxin is released and active in the bacterial environment. Therefore, a vaccine targeting this toxin is expected more to neutralize its deleterious effects than to have direct effects on the bacterium. To illustrate this statement, we have demonstrated in the present work in a lethal challenge with biologically active Ply that anti-dPly antibodies were very efficacious at neutralizing the toxin, whereas in another work only trends of protection were observed in dPly-immunized mice when they were challenged with the full pathogen in a murine model of lung colonization (data not shown). In the latter work, the anti-dPly immune responses appeared not sufficient to provide full protection against pneumococcal infection. Nevertheless, the demonstration of the in vivo neutralization of the toxic effect of Ply is a relevant observation with respect to the pneumococcal pathogenesis,^22^ and supports the use of dPly as vaccine antigen.

Bacterial colonization of the nasopharynx is the first and necessary step before infection takes place. Therefore, protection against colonization would help to contain pneumococcal spread and thus subsequent pneumococcal disease. In this respect, Ply is an antigen of choice, as it has recently been shown to play a role in the establishment of long-term carriage by induction of the immunomodulatory TGFβ1^18^. With a mouse model, we could show that intranasal immunization with dPly was protective against nasopharyngeal colonization. However, some animals were still colonized by the pathogen on the latest time point. This may indicate that a longer time was needed for some mice to clear bacterial colonization or that some were non-responsive to immunization. We did not address this question by investigating a longer time course since the effect of dPly immunization was already demonstrated with the 2 chosen time points, which was the goal of the study. In addition, the clinical relevance of an incomplete protection against colonization in a mouse model is unknown, considering the differences between challenges in mice with a defined amount of bacterium on the one hand and the occurrence of a natural pneumococcal infection in humans on the other hand.

Protection in the nasopharynx was observed not only against strains with homologous allele 1, but also against a strain with the non-homologous allele 2, which suggest cross-protection to other alleles than the vaccine allele. There are 18 known alleles of Ply,^24^ but alleles 1 and 2, differing by one amino acid are by far the most prevalent ones. Only by protecting against these 2 alleles dPly vaccination would cover about 85% of the circulating pneumococcal strains,^24^ and it cannot be excluded that cross-protection to other alleles is possible, extending coverage even further, which remains to be investigated. These data on nasopharyngeal colonization highlights the possible dual aspect of the immune response to *S. pneumoniae*. When given intra-nasally, as was the case of the nasopharyngeal colonization model, pneumococcal antigens are suspected to elicit Th17-dependent, antibody-independent mechanisms of protection,^40,41^ whereas intramuscular injection of an alum-adjuvanted formulation favors the production of antibodies. Induction of the IL17 pathway allows the monocyte/macrophage clearance of the first step of pneumococcal infection,^42^ while antibodies may be effective at later stages. The role of Th17 in this clearance mechanism needs to be investigated further, but nevertheless, these observations suggest that it may be beneficial that protein-based vaccination against pneumococcus induces both humoral and cellular arms of the immune system, in order to act at different levels of pneumococcal infection.

Overall, we have shown that a stable chemically detoxified Ply antigen can be obtained and that immunization of animals with this modified antigen elicits immune responses that are able to inhibit naso-pharyngeal colonization and to neutralize the toxin. These results supported the inclusion of dPly in a pneumococcal vaccine. Further experiments, combining dPly with the pneumococcal histidine triad D protein (PhtD)^43^ showed protection against disease in a non-human primate model of pneumonia.^44^ In that work, a statistical model helped to evaluate the contribution of each antigen and calculated the association between the levels of anti-Ply antibodies and protection. A concentration of anti-Ply antibodies ≥5 µg/ml was found associated with 80% chance of surviving the challenge, and with a concentration ≥ 116 µg/ml, the survival rate was 95%. In the protected primates, a reduction in bacterial load was noticeable within the first day after inoculation, showing the necessity to neutralize the pathogen very rapidly to survive the challenge. In this regard, the role of the anti-Ply antibodies was probably essential as Ply is an important pneumococcal factor in the early pathogenesis of pneumonia.^45^

Based on the results of the present and other works, dPly is currently in early clinical development in association with PhtD, as protein-only vaccine formulations^46,47^ or combined with pneumococcal conjugates.^48^

## Materials & methods

### Bacteria

The serotype 2 (strain D39, Ply allele 1) was kindly provided by JC Paton (University of Adelaide, Australia). The serotypes 6B and 4 (CDC strains, Ply allele 1 and 2, respectively) were obtained from the Centers for Disease Control and Prevention (CDC) and serotype 6B (strain 493/73, Ply allele 1) from the Statens Serum Institut (SSI, Denmark).

### Animals

OF1 female mice used in this study were purchased from Charles River laboratories (Lyon, France). Balb/c mice were from Harlan (Horst, The Netherlands). OFA male rats (8-10-week old, Charles River, France) were used for the evaluation of *in vivo* dPly reactogenicity. All animal studies were ethically reviewed and carried out at GSK Vaccines (Rixensart, Belgium) in accordance with European Directive 86/609/EEC or European Directive 2010/63, and the GSK Policy on the Care, Welfare and Treatment of Animals.

### Production and chemical detoxification of Ply

The Ply gene (allele 1) was cloned from the *S. pneumoniae* 6B 493/73 strain, recombinantly expressed in *Escherichia coli* and purified through multiple steps including hydrophobic interaction chromatography, denaturation, refolding and filtration. These different steps were optimized to yield a biologically active recombinant Ply. Purity and restoration of biological activity after refolding were ascertained by SDS-polyacrylamide gel electrophoresis (SDS-PAGE), anti-host cell proteins immunoblotting, hemolysis assay, mouse intranasal lethal challenge and enzyme-linked immunosorbent assay (data not shown).

Detoxification of Ply was achieved by formaldehyde treatment in the presence of lysine. Lysine was incorporated to the mixture to partially quench formaldehyde and thereby avoid or limit cross-linking of Ply molecules. In a pilot phase, different formaldehyde concentrations (0.05, 0.075 or 0.1% w/v), lysine concentrations (25, 37.5 or 50 mM), pH (7.0 or 7.2), incubation times (7, 14, 21 or 28 days), and combinations thereof were assessed to determine the best combination leading to a fully detoxified and immunogenic molecule. The reversibility of detoxification was also taken into account for the pilot conditions, which was assessed through toxicity testing after incubation of dPly samples at 37°C for up to 35 d.

The final retained conditions for detoxification were incubation for 21 d at 40°C in potassium phosphate 25 mM, pH 7.0, containing formaldehyde 0.1% (w/v) and lysine 50 mM. Clarification, concentration, diafiltration (30 kDa) and sterilization by filtration were performed after detoxification. Purified dPly was kept at −70°C until use.

### In vivo reactogenicity and toxicity

Rats (n = 3) received intramuscular injections (one in each tibialis) of 10 µg Ply or dPly in 50 µl PBS 0.15 M, pH 6.8. Control injections consisted of vehicle only. At day 3 post-injection, the animals were sacrificed and tibialis samples at the injection site were taken. Samples were fixed in 10% formaldehyde in PBS, and cut into 2 mm-thick pieces. These pieces were dehydrated and embedded in paraffin from which 7 µm-thick slices were obtained. The slices were stained with the Masson's trichrome method and examined microscopically.

In another model to evaluate dPly toxicity, OF1 female mice (4 weeks-old; n = 10/group) were challenged intranasally with either PBS, 10 µg Ply, or 50 µg dPly. Mortality was recorded during the following 3 d.

### SDS-PAGE and immunoblotting

Samples were boiled during 5 min in Tris sample buffer with β-mercaptoethanol and run (8-10 µg/lane) in 4-20% Criterion gels (Biorad). After migration, gels were stained with Coomassie Brilliant blue G250 or used in immunoblotting.

For immunoblotting, nitrocellulose membrane blots were probed after protein transfer with in-house monoclonal mouse anti-Ply antibodies or polyclonal rabbit anti-Ply antibodies. Mouse antibodies were detected by biotin-labeled sheep anti-mouse Ig antibody (Amersham, RPN 1001V1) and rabbit antibodies by biotin-labeled donkey anti-rabbit Ig antibody (Amersham, RPN 1004V1). In both cases, the bands were revealed by addition of streptavidin-horseradish peroxidase complex (Amersham, RPN 1051V) followed by incubation in 4-chloro-1-naphtol in the presence of hydrogen peroxide.

### ELISA for the detection of anti-Ply antibodies

ELISA microtiter plate was coated with Ply. After washing, a reference serum and twofold serial dilutions of the mouse sera in PBS-Tween-20 0.05% were added to the microtiter plate and incubated for 30 min at room temperature. After another washing step, peroxidase-conjugated goat anti-mouse IgG (Jackson Immunoresearch; 1/2500) was added into the wells for a 30-min incubation at room temperature. Colorimetric detection was achieved by addition of the peroxidase substrate o-phenylenediamine, and the plate was read in a microplate reader at 490 nm. The individual IgG concentrations (expressed as µg/ml) were calculated by the 4-parameter method using the Soft Max Pro software.

### Hemolytic activity and hemolysis inhibition assay

To evaluate hemolytic activity, 2-fold serial dilutions of the Ply to be tested were pipetted (100 µL) into a microtiter plate with U- bottoms. An equal volume of sheep erythrocytes (1% vol/vol) was added to the wells and the mixture was incubated for 30 min at 37°C. After centrifugation, the supernatants were transferred to another plate and the optical density was read at 405 nm to measure the extent of erythrocytes lysis. Hemolytic activity was defined as the concentration of Ply leading to the lysis of 50% of erythrocytes.

For the hemolysis inhibition assay, OF1 female mice (4 weeks-old; n = 20/group) were immunized intramuscularly (i.m.) at day 0 and 14 with 1 µg dPly adsorbed on 50 µg AlPO_4_ (total volume: 50 µl). Control animals were vaccinated with AlPO_4_ only. Sera were collected on day 27, and cholesterol was removed from sera by chloroform treatment.

For the assay, serum pools were serially diluted 2-fold and 100 µL of each dilution added to a microtiter plate. Next, 4 hemolysis unit of Ply was added to each well for a 15-min incubation at 37°C. One HU is the amount in ng/ml able to lyse 50% of erythrocytes. After incubation with Ply, 100 µL of sheep erythrocytes suspension (1% vol/vol) was added to each well and the mixture was incubated for 30 min at 37°C. After centrifugation, the extent of lysis was measured in a spectrophotometer at 405 nm. Results were expressed as the serum dilution that inhibited 50% of Ply lytic activity.

### Mouse Ply lethal intranasal challenge

OF1 female mice (4 weeks-old; n = 20/group) were immunized intramuscularly (i.m.) at day 0 and 14 with 1 µg dPly adsorbed on 50 µg AlPO_4_ (total volume: 50 µl). Control animals were vaccinated with adjuvant only. At day 28, mice were challenged intranasally with 2 µg Ply (equivalent to 1670 HU). The mortality was recorded during 3 d after challenge.

### Mouse pneumococcal nasopharyngeal colonization model

For the nasopharyngeal colonization model, Balb/c mouse strain was used. The mice (4 weeks-old; n = 10/time-point) were immunized at days 0, 14 and 28 by the intranasal route with 10 µg of dPly supplemented with 0.5 µg of *E. coli* heat-labile enterotoxin (LT) as an adjuvant (except in the last immunization). Control animals (4 weeks-old; n = 10/time-point) were vaccinated with adjuvant only. At day 42, mice were challenged intranasally with 2 × 10^5^ cfu/10 µl of type 6B/CDC or type 2/D39 strain, both strains having a Ply of the the same allele as dPly (allele 1). Challenge was also performed with 4/CDC strain, carrying heterologous Ply (allele 2). The challenges were performed using a small bacterial inoculum volume (2 × 10^5^ cfu in 10 µl in one nostril). Bacterial colonies (CFU numbers) were counted in nasal washings collected 2 and 4 d after the challenge (2 and 6 d after challenge for 4/CDC). Only CFUs in nasal washings were taken into account as our internal records have shown that measuring CFUs in nasal washings is relevant, reproducible, and proportional to measurements of CFU encompassing both nasal washings and excised nasopharyngeal tissue homogenates.

### Statistical analyses

Survival data were analyzed with a 2-sided Fischer's exact test (comparison of proportions on Day 3). All colony counting data, after normalization, were compared with ANOVA, followed by the Dunnett post-test when ANOVA was found significant.
